# Evaluation of health system readiness and coverage of intermittent preventive treatment of malaria in infants (IPTi) in Kambia district to inform national scale-up in Sierra Leone

**DOI:** 10.1186/s12936-021-03615-3

**Published:** 2021-02-06

**Authors:** Maria Lahuerta, Roberta Sutton, Anthony Mansaray, Oliver Eleeza, Brigette Gleason, Adewale Akinjeji, Mohamed F. Jalloh, Mame Toure, Getachew Kassa, Steven R. Meshnick, Molly Deutsch-Feldman, Lauren Parmley, Michael Friedman, Samuel Juana Smith, Miriam Rabkin, Laura Steinhardt

**Affiliations:** 1 ICAP at Columbia University, Mailman School of Public Health, New York, USA; 2 Department of Epidemiology, Mailman School of Public Health, Columbia University, New York, USA; 3 ICAP at Columbia University, Freetown, Sierra Leone; 4 United States Centers for Disease Control and Prevention, Freetown, Sierra Leone; 5 Immunization Systems Branch, Global Immunization Division, Center for Global Health, Centers for Disease Control and Prevention, Atlanta, USA; 6 Department of Epidemiology, Gillings School of Global Public Health, University of North Carolina, Chapel Hill, NC USA; 7 National Malaria Control Program, Ministry of Health and Sanitation, Freetown, Sierra Leone; 8 Malaria Branch, Division of Parasitic Diseases and Malaria, Center for Global Health, Centers for Disease Control and Prevention, Atlanta, USA

**Keywords:** Malaria, Sierra Leone, Infants, IPTi, Evaluation, Coverage, Household survey, National scale-up

## Abstract

**Background:**

Intermittent preventive treatment of malaria in infants (IPTi) with sulfadoxine-pyrimethamine (SP) is a proven strategy to protect infants against malaria. Sierra Leone is the first country to implement IPTi nationwide. IPTi implementation was evaluated in Kambia, one of two initial pilot districts, to assess quality and coverage of IPTi services.

**Methods:**

This mixed-methods evaluation had two phases, conducted 3 (phase 1) and 15–17 months (phase 2) after IPTi implementation. Methods included: assessments of 18 health facilities (HF), including register data abstraction (phases 1 and 2); a knowledge, attitudes and practices survey with 20 health workers (HWs) in phase 1; second-generation sequencing of SP resistance markers (pre-IPTi and phase 2); and a cluster-sample household survey among caregivers of children aged 3–15 months (phase 2). IPTi and vaccination coverage from the household survey were calculated from child health cards and maternal recall and weighted for the complex sampling design. Interrupted time series analysis using a Poisson regression model was used to assess changes in malaria cases at HF before and after IPTi implementation.

**Results:**

Most HWs (19/20) interviewed had been trained on IPTi; 16/19 reported feeling well prepared to administer it. Nearly all HFs (17/18 in phase 1; 18/18 in phase 2) had SP for IPTi in stock. The proportion of parasite alleles with *dhps* K540E mutations increased but remained below the 50% WHO-recommended threshold for IPTi (4.1% pre-IPTi [95%CI 2–7%]; 11% post-IPTi [95%CI 8–15%], p < 0.01). From the household survey, 299/459 (67.4%) children ≥ 10 weeks old received the first dose of IPTi (*versus* 80.4% for second pentavalent vaccine, given simultaneously); 274/444 (62.5%) children ≥ 14 weeks old received the second IPTi dose (*versus* 65.4% for third pentavalent vaccine); and 83/217 (36.4%) children ≥ 9 months old received the third IPTi dose (*versus* 52.2% for first measles vaccine dose). HF register data indicated no change in confirmed malaria cases among infants after IPTi implementation.

**Conclusions:**

Kambia district was able to scale up IPTi swiftly and provide necessary health systems support. The gaps between IPTi and childhood vaccine coverage need to be further investigated and addressed to optimize the success of the national IPTi programme.

## Background

Despite advancements in malaria prevention and control, there were an estimated 229 million malaria cases and 409,000 malaria deaths in 2019 [1], highlighting the need for additional interventions to reduce the malaria burden. In 2010, the World Health Organization (WHO) recommended intermittent preventive treatment of malaria in infants (IPTi) with sulfadoxine-pyrimethamine (SP), as an intervention to reduce malaria incidence and its complications in infants [2]. In areas with low levels of SP resistance, the WHO recommends that infants receive three doses of IPTi with SP at 10 weeks, 14 weeks, and 9 months of age, as part of the routine vaccination schedule of the Expanded Programme on Immunization (EPI). Seven randomized clinical trials have shown that IPTi can reduce malaria morbidity by 30%, all-cause hospitalizations by 23%, and anaemia by 21%, with no severe adverse reactions or decreased efficacy of co-administered EPI vaccines [3–9].

IPTi with SP is recommended for countries (or sub-national areas) with moderate-to-high malaria endemicity where SP resistance levels are not high (defined as ≤ 50% prevalence of the *Plasmodium falciparum* enzyme dihydropteroate synthetase *(dhps)* gene K540E mutation) [2]. Although many countries in sub-Saharan Africa meet these criteria, to date Sierra Leone is the first and only country to implement IPTi on a large scale, first in two pilot districts and then scaling up nationwide over 15 months.

## Malaria in Sierra Leone

Malaria is endemic in Sierra Leone, with stable and perennial transmission throughout the country. Over 85% of malaria cases in Sierra Leone are attributed to *P. falciparum*. Malaria remains the leading cause of both disability-adjusted life years (DALYs) and years of life lost (YLL) due to premature death in Sierra Leone [10], with more than 2.6 million confirmed cases of malaria reported in 2019 [1]. The National Malaria Control Programme (NMCP) has overseen substantial achievements in malaria prevention efforts, with mass insecticide-treated bed net (ITN) distribution increasing the proportion of children under five years sleeping under ITNs from 2% in 2001 to 44% in 2016 [11]. Despite these achievements, malaria continues to be the leading cause of morbidity and mortality in children under five in Sierra Leone; approximately 40% of children under five and almost 25% of infants were found to be parasitaemic in a national household survey in 2016 [11, 12].

## Implementation of IPTi in Sierra Leone

In 2015, the NMCP formally added IPTi with SP to the 2016–2020 National Malaria Strategic Plan in recognition of the need to aggressively combat malaria. In 2017, with support and technical guidance from partners and donors, the NMCP launched IPTi within the existing EPI delivery platform for child immunization services. Implementation was rolled out in two pilot districts, Kambia and Pujehun, in March 2017 and two additional districts, Kenema and Western Area Urban, in August 2017 before national scale-up in the remaining 10 districts in three waves in 2018. An IPTi Task Force was created to guide strategic planning activities, including the development of an Implementation Field Guide, adapted from the WHO Global Malaria Programme [13], adaptation of the EPI register and child health cards to include IPTi, forecasting and quantification of SP stock needs, and development of a competency-based training curriculum.

Trainings were designed in a cascading manner, with an initial national-level training of trainers for 40 participants, including NMCP representatives and District Health Management Teams (DHMT) from Kambia and Pujehun. This was followed by district-level trainings for up to two staff members from each health facility. Interactive training sessions were designed to teach participants about IPTi; foster and practice skills on messaging IPTi to other health workers (HWs), caregivers, and community members; reinforce appropriate dosage and delivery of SP; and demonstrate correct completion of monitoring and reporting tools. At the end of the district training, staff received the first allotment of SP stocks and materials (spoons, cups, and updated child health cards and registers) for their health facility.

Supporting materials such as posters, fliers, job aids, monitoring and evaluation (M&E) tools, and social and behaviour change materials were also developed and distributed to all health facilities. A community education strategy that included radio spots introducing IPTi was launched prior to implementation in Kambia and Pujehun. Additionally, as part of a coordinated effort by the NMCP and the DHMTs, IPTi supportive supervision checklists were developed and implemented during supervisory visits conducted at HF to monitor IPTi implementation in the pilot districts.

To inform the national scale-up of IPTi and to document lessons learned from the first, and only, national IPTi programme, an external evaluation was conducted. The evaluation assessed the quality, acceptability, and coverage of IPTi pilot activities in Kambia to provide insights into successes, challenges, and areas for improvement.

## Methods

### Study overview

A mixed-methods evaluation was conducted in Kambia with two phases of data collection. Phase 1 took place in March 2017, pre-IPTi implementation; and July 2017, three months after the launch of IPTi implementation. Phase 2 took place July–September 2018, 15–17 months after IPTi implementation.

### Study setting

The evaluation took place in the district of Kambia, the first to implement IPTi. Kambia district is located in northern Sierra Leone bordering Guinea and has a population of more than 340,000 inhabitants, most of whom are farmers. In 2017, Kambia had 69 public health facilities, including one district-level hospital, 15 community health centres, 16 maternal and child health posts, and 37 community health posts. In Kambia, according to the 2016 Malaria Indicator Survey, 48.7% of children under 5 had slept under an ITN the previous night, 48.3% of children 6–59 months were positive for malaria via microscopy and 86.6% of women 15–49 years received two or more doses of SP in the last pregnancy [11]. The Ministry of Health and Sanitation distributed mosquito bed nets in 2017 across the country, including Kambia, as part of the Ministry of Health Malaria Control Strategic Plan 2016–2020.

### Data collection

**Fig. 1:**
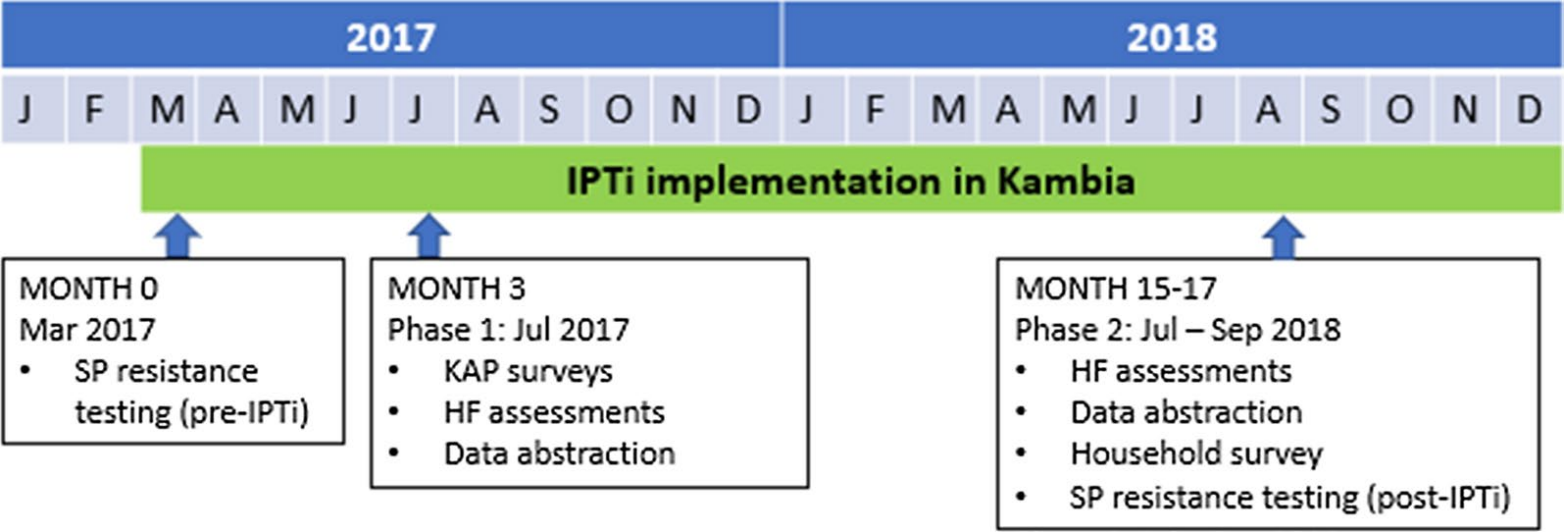
Timing of evaluation activities

Data collection methods included a mix of: (a) health facility (HF) assessments; (b) a knowledge, attitudes, and practices (KAP) survey with HWs; (c) HF register data abstraction; (d) a household survey; and (e) analysis of molecular markers of SP resistance. The timing of the various data collection methods is presented in Fig. 1.

### HF assessments

HF assessments, conducted in both phase 1 and phase 2 with purposively recruited persons in-charge, vaccinators, and laboratory/pharmacy staff, captured HF characteristics including size, patient volume, staffing, number of HWs trained in IPTi, and availability of SP, equipment, supplies, and IPTi monitoring tools. HFs were selected among those providing immunization services using stratified simple random sampling by HF type. A total of 18 HFs were included in the sample, representing a quarter of all HFs in Kambia district. The same HFs were visited in phases 1 and 2.

### KAP survey

The KAP survey was conducted in phase 1 with purposively recruited HWs who were providing IPTi to eligible infants at HF selected for the HF assessment. After obtaining written consent, trained interviewers conducted the KAP survey in English or Krio using a tablet-based survey. The knowledge domain of the questionnaire consisted of multiple-choice questions designed to assess fundamental knowledge of IPTi. The attitudes and practices domains used single-selection closed-ended questions to explore respondents’ impressions of the IPTi program and its acceptability to HWs and families, and their practices when administering IPTi to eligible infants.

### HF data abstraction

As part of the HF assessments in phase 1 and 2, routine data on malaria cases, and vaccine doses and IPTi doses administered were abstracted from the outpatient and EPI registers and monthly summary forms at the selected HFs. Each abstraction was conducted in both phase 1 and phase 2 and included data for the previous 13–14 months (from April 2016-June 2018).

### Household survey

In phase 2, a household survey was conducted with caregivers of children 3–15 months old to assess perceptions, acceptability, and coverage of IPTi. Households were selected using a two-stage random selection process. At the first stage, 44 enumeration areas (EAs) were randomly selected from the 2015 Sierra Leone census list using simple random sampling, stratified by urban or rural geographic setting. Within each selected EA, all households were mapped and enumerated, generating a household listing with information on the age and sex of all household members. Then, simple random sampling was used to select 10 eligible households (at least one caregiver 18 years or older of a child aged 3–15 months) per EA. In EAs that had less than 10 eligible households, all eligible households were selected in EAs. Assuming a non-response rate of 10% and a design effect of 2 (given clustering of children within chiefdoms and unknown intracluster correlation coefficient), a sample size of 440 households was needed to have sufficient precision to determine an IPTi coverage of 50% with a ± 7-percentage point margin of error (95% CI 43–57%) among children aged 3–15 months.

Survey teams visited selected eligible households, identified all eligible child(ren)’s caregiver(s), and introduced the purpose of the survey. After obtaining written informed consent, the survey team administered the household questionnaire to the caregiver(s) of all eligible children in the household in their preferred language (English, Krio, Temne, Susu or Limba). If the caregiver was not home at the time of the initial visit, the survey team returned to the household three times on different days. Survey questions were entered by interviewers in a tablet programmed using SurveyCTO (Dobility, Inc., Massachusetts, USA), based on Open Data Kit platform and included questions on household characteristics; literacy and education of caregivers; awareness of health messages; health-seeking behaviours; knowledge of malaria; ownership/use of ITNs; knowledge of IPTi services; experiences with IPTi services/receipt of IPTi by eligible children; health status/recent illness or hospitalization; and child immunization history. To measure coverage of IPTi and other routine immunizations, data were abstracted from available child health cards. Similar to the approach used on the Sierra Leone Demographic and Health Surveys [14], when child health cards were unavailable, caregivers were asked to recall receipt of IPTi and routine immunizations.

### SP resistance testing

At two separate HFs in Kambia not included in the HF assessment, verbal consent was obtained from caregivers of children < 7 years and from adults ≥ 18 years presenting to the outpatient department and being tested for malaria before IPTi implementation (pre-IPTi in March 2017) and approximately one year later (post-IPTi in May 2018). Among those consenting, the same finger prick was used to conduct the malaria rapid diagnostic test as well as to collect a sample of blood on Whatman 3MM chromatography paper. These dried blood spots (DBS) from malaria-positive individuals were retained and were shipped to the Infectious Disease Epidemiology and Ecology laboratory at the University of North Carolina, USA. DNA was extracted from the DBS using Chelex extraction in pools of 10 DBS per pool and prepared for amplicon deep sequencing [15, 16]. Sequencing focused on the A437G, K540E, and A581G mutations of the *P. falciparum dhps* gene and N51I, C59R, and S108N mutations of the *dhfr* gene, which have been shown to confer resistance to sulfadoxine and pyrimethamine, respectively [17, 18]. The combination of the triple dihydrofolate reductase *(dhfr)* gene (N51I, C59R, and S108N) and the double *dhps* (A437G, K540E) mutations collectively form the quintuple mutation [19] which confers high-level SP resistance and is a significant predictor of SP *P. falciparum* treatment failure [20, 21]. In addition, the A581G mutation has emerged alongside the quintuple mutations at high levels in East Africa, and is associated with high rates of SP clinical failure when the mutation prevalence is above 10% in pregnant women receiving IPT [22]. The pooled analysis used in this paper employs second-generation sequencing to quantify mutant allele frequencies in the *dhps* and *dhfr* genes to generate estimates of the prevalence of key mutations among the parasite population found in Kambia at each time point. Sequencing data was obtained using molecular inversion probes (MIP), a novel technology for high-throughput deep sequencing of *Plasmodium* species [23–25]. Individual MIP probes were designed to flank the *dhps* and *ASTR* [23]. Samples from each clinic and each time point were run in duplicate.

### Data analysis

Descriptive analyses were conducted for data from the HF assessments, KAP survey, and household survey in SAS v 9.4 (Cary, NC, USA). For the molecular analyses, differences in the proportions of resistant alleles were compared using Fisher's exact tests using the R statistical platform v 3.6. For the household survey, sample means/proportions for measures of interest and their 95% confidence intervals were estimated and weighted for unequal sampling probabilities and non-responses and further adjusted for clustering of respondents using the ‘svy’ commands in Stata 14.0 (College Station, Texas, USA) and the Rao-Scott correction for chi-square tests [26]. Three binary composite variables were calculated to capture vaccine dose uptake verified in the child health card or through caregiver-recall (when the card was unavailable). The first was for doses scheduled at 10-weeks (first dose of IPTi and second dose of pentavalent vaccine [a combination vaccine that protects against Diphtheria, Pertussis, Tetanus, Hepatitis B and Hib]). The second was for doses scheduled at 14-weeks (second dose of IPTi and third dose of pentavalent vaccine) and the third was for doses scheduled at 9-months (third dose of IPTi and first dose of measles vaccine). Complete IPTi coverage was defined as receiving three IPTi doses (IPTi 1, 2 and 3) among children ≥ 9 months and receiving two doses (IPTi 1 and 2) among children ≥ 14 weeks. All household survey results presented in this report are weighted, unless indicated otherwise. Confidence intervals for the SP resistance markers were calculated using the binomial ‘exact’ method.

Interrupted time-series analysis using a Poisson regression model and generalized estimating equations with robust variance estimation was used to analyze confirmed malaria cases among patients < 12 months based on abstracted HF register data. Covariates in the model included the total number of outpatient visits among patients < 12 months, the proportion tested for malaria, and a dummy variable for high malaria transmission season (July – October).

## Results

### HF assessment

The 18 sampled HFs included 1 clinic for children under five years of age at the district hospital, 4 community health centres, 3 community health posts, and 10 maternal and child health posts. Nearly all HFs (17/18 in phase 1 and 18/18 in phase 2) had SP for IPTi in stock (Table 1). However, fewer HFs reported having a system of estimating SP supply needs in phase 2 versus phase 1 (11/18 in phase 1 and 6/18 in phase 2). Availability of improved drinking water sources for IPTi administration at HFs, defined as chlorinated well water, sachet water and water purified with tablets, increased over time but still had gaps (7/18 in phase 1 and 11/18 in phase 2). Over time, fewer HF had revised child health cards that allowed for recording receipt of each dose of SP for IPTi (15/18 in phase 1 *versus* 12/18 in phase 2). No change was seen in the number of HFs providing IPTi through outreach services at least once per week (8/18) or in those providing IPTi for free (18/18). Most (15/18 in phase 1 and 18/18 in phase 2) HFs reported having supervisory visits in the last six months that addressed IPTi provision.

**Table 1:** Health facility assessment results on IPTi supplies, service provision, and supervisory visits in Phase 1 and 2

	Phase 1		Phase 2	
	N (n = 18)	%	N (n = 18)	%
Stocks and supplies				
SP for IPTi in stock	17	94.4	18	100.0
System of estimating needed SP supply established	11	61.1	6	33.3
Improved drinking water for IPTi administration available^a^	7	38.9	12	66.7
Revised child health cards for IPTi documentation available	15	83.3	12	66.7
Service provision				
IPTi provided through outreach services	18	100.0	18	100.0
2–3 times a week	4	22.2	1	5.6
Once a week	4	22.2	7	38.9
Other less frequent	10	55.6	10	55.6
IPTi provided at no cost to patients	18	100.0	18	100.0
Supervisory visit covering IPTi provision	15	83.3	18	100.0

^a^Improved drinking water included chlorinated well water, sachet water and water purified with water. Unimproved drinking water included untreated and not boiled well water, tap water and nearby pond

### KAP survey

KAP surveys were conducted with 18 maternal and child health (MCH) aides, one community health assistant, and one immunization officer at 18 HFs. Among the 19/20 HWs trained on IPTi, most rated the didactic training provided by the DHMT as good (15/19) or excellent (3/19) quality and felt confident in providing IPTi services (16/19) as a result of training. Results of the KAP survey indicated a good understanding of IPTi among HWs. Of 20 respondents, 15 scored at least 80% on the 20-question knowledge assessment (see Additional file 1).

According to HWs, most eligible infants received IPTi; 17/20 HWs reported administering IPTi to all eligible infants they saw in the past week. Acute illness was the only reason HWs provided for not administering IPTi to eligible infants. When asked, 6/20 HWs reported that they had witnessed an infant prescribed SP vomiting the drug immediately after receiving it but indicated this occurred rarely.

HWs did not report any barriers related to community acceptance of IPTi. HWs reported that most parents, caregivers, and community leaders accepted IPTi services: 19/20 reported that no parents or caregivers refused IPTi services when offered, and 19/20 somewhat agreed to strongly agreed that community leaders were supportive of IPTi.

## Household survey

### Study population

Surveys were completed for 433 of 440 sampled households (response rate of 98.3%). Among identified caregivers (449), all but one were women, the mean age was 26.2 years, and 90% were married (Table 2). The majority (71.7%) had no schooling and 79.8% were not able to read at all. Data were collected for 459 infants (22 households had 2 eligible children and 3 households had 3 eligible children).

**Table 2:** Demographic characteristics of caregivers participating in household survey during Phase 2

	N	% (weighted)
Total # of caregivers	449	
Caregiver sex		
Male	1	0.2
Female	448	99.8
Age at interview (years), mean (std)		26.2 (7.15)
18–24	204	47.5
25–29	101	24.7
30–34	70	16.4
35–39	32	7.6
40 +	16	3.8
Marital status		
Currently married	398	90.2
Currently living with partner	22	4.7
Widowed	9	1.6
Divorced/separated	3	0.6
Single	17	2.9
Number of children given birth to (mean [IQR])		3 (2–5)
Number of children given birth to that are still alive (mean [IQR])		3 (2–4)
Highest level of education		
No schooling	307	71.7
Primary	71	15.1
Junior/Senior Secondary	66	12.3
Diploma/Vocational	5	0.9
Literacy		
Cannot read at all	348	79.8
Able to read only parts of sentence	57	11.4
Able to read whole sentence	44	8.9
Religion		
Muslim	424	95.0
Christian	23	4.7
None	2	0.4

### Malaria prevention and treatment

Caregivers reported that most of their children (88.5%) slept under an ITN the previous night (Table 3). Among the 103/459 (22.7%) children that had reported a fever in the previous two weeks, 50.5% (55/103) were diagnosed with confirmed malaria, according to caregiver. Among the 81/103 (79.3%) children whose caregivers who sought advice for the fever, 13/81 (17.2%) were hospitalized and 77/81 (95.3%) were given medication for the illness (with or without prescription).

**Table 3:** Malaria prevention, illness in past two weeks, and IPTi coverage and perceptions from household survey in Phase 2

	n	% (weighted)	95% confidence interval
Among children (N = 459)			
Slept under mosquito net previous night	408	88.5%	84.5—92.6
Ill with fever in previous 2 weeks	103	22.7%	17.0—28.4
Blood taken from finger or heel	66	65.3%	52.5—78.2
Diagnosed with malaria			
Yes	55	50.5%	39.4—61.6
No	45	47.6%	36.3—58.9
Don't Know	3	1.9%	0—4.7
Sought advice for illness of child	81	79.3%	69.7—88.9
Among those that sought advice, child hospitalized for this illness	13	17.2%	6.4—28.0
Took medication for illness	77	95.3%	90.6—100
Received IPTi-1^a^	299	67.4%	58.3 – 75.3
Reasons for not receiving IPTi among those with no ITPi (n = 114)			
Did not know it was required	42	34.7	(21.9, 50.2)
Child not offered medication by provider	15	14.4	(8.2, 24.1)
Has not visited a facility for immunization since birth	11	10.5	(4.8, 21.6)
Time/place of immunization not convenient	8	6.2	(3.2, 11.8)
Feared side effects	7	5.9	(2.6, 12.7)
Had to pay	7	5.0	(1.8, 13.3)
Medication was not at the facility	6	5.5	(2.1, 13.8)
Distance/Lack of or cost of transport	4	3.7	(1.1, 12.0)
Did not know where to get it	3	2.9	(0.9, 9.0)
Cultural/religious reasons	2	2.2	(0.1, 9.3)
Needed to work	2	1.7	(0.4, 7.5)
Medication affects other vaccinations	1	0.6	(0.1, 4.7)
Among caregivers (N = 449)			
Heard about a new way to prevent infants from getting malaria	296	67.4%	59.7—75.1
Heard about a new malaria tablet (among those who heard about a new way to prevent malaria among infants)	289	97.9%	96.3—99.6
Perceived the new malaria tablet as very safe	279	96.1%	93.6—98.6

^a^According to card and mother’s recall; 40 children who only received IPTi-2 and/or IPTi-3 according to their vaccination card were not counted as receiving IPTi-1

^b^The total does not equal 100% because it was a multiple choice question

Among caregivers, 67.4% reported they had heard about a “new way to prevent infants from getting malaria,” of whom 97.9% were specifically aware of a “new tablet to prevent malaria in infants.” Of these, 96.1% believed the “new malaria preventative tablet for infants” was very safe. Among the 296 caregivers who heard messages on “a new way to prevent infants from getting malaria”, the most common source of information was government clinic/hospital (84.5%), followed by community health workers (67.4%) and radio (25.4%).

### IPTi and vaccine coverage

**Fig. 2:**
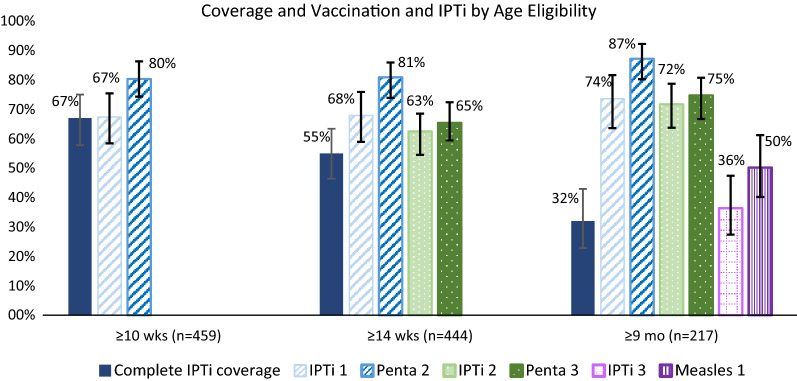
Coverage of IPTi dose and vaccination by age eligibility among children in Kambia. Complete IPTi coverage was receiving the appropriate doses for that age group. There were 40 children who only received IPTi doses 2 and/or IPTi dose 3 according to their vaccination card (missed IPTi dose 1). Error bars represent 95% confidence interval

Child health cards were available and verified for 395/459 (86.2%) of the sampled children. Vaccination status was determined based on child health card for 395 (86.2%) and caregiver report for 64 (13.8%) who did not have a card. Among children ≥ 10 weeks old (n = 459), 67.4% had received the first dose of IPTi, and 80.3% received the second pentavalent dose, given at the same time); among children ≥ 14 weeks old, 62.5% had received the second dose of IPTi and 65.4% received the third pentavalent dose, given at the same time. Among children ≥ 14 weeks (n = 444), 54.9% received IPTi-1 and IPTi-2. Among children ≥ 9 months old (n = 217), 36.4% had received the third dose of IPTi and 52.2% had received the first measles vaccination, given at the same time (Fig. 1). Among children ≥ 9 months, 32.2% received all three doses (IPTi-1, 2 and 3), considered complete IPTi coverage (Fig. 2).

Among those with a child health card, median age for first IPTi dose was 12.2 weeks (2.2 weeks median delay), for the second dose was 17.4 weeks (3.4 weeks median delay), and the third dose was 9.3 months (~ 1.2 week median delay), similar to other routine vaccines co-administered with IPTi.

Overall, 138 (29.2%) surveyed children did not receive any IPTi. When caregivers whose children had never received IPTi were asked for the reasons why, they most frequently cited that they did not know IPTi was required (34.7%), their child was not offered IPTi (14.4%), or their child had not visited a facility for immunizations since birth (10.5%). Other reasons cited were lack of convenient times/places for IPTi, fear of side effects, distance or inability to pay transportation and/or facility costs, and lack of SP at the facility (Table 3).

In bivariate analysis, household wealth, caregiver schooling, and caregiver literacy were not significantly associated with complete IPTi coverage among children ≥ 14 weeks of age (i.e., receiving IPTi doses 1 and 2), nor was ITN usage among infants (Table 4). However, children of caregivers who had heard of or saw materials about a new way to prevent infants from getting malaria were significantly more likely to have received both IPTi-1 and IPTi-2 doses compared to those whose caregivers were not aware of this new prevention method (64.5% *versus* 35.0%, p < 0.001) (Table 4).

**Table 4:** Factors related to receipt of IPTi complete coverage (both IPTi-1 and IPTi-2) among infants ≥ 14 weeks (n = 444)

Variable	Complete IPTi-1/2 coverage, n/N, (%)		p-value from Chi-square test^a^
Caregiver schooling			
Ever attended	76/139	(53.7)	0.826
Never attended	159/305	(55.4)	
Caregiver literacy			
None	183/350	(55.0)	0.820
Partial	29/54	(51.3)	
Full	23/40	(58.9)	
Caregiver heard/saw a new way to prevent infants from getting malaria			
Yes	184/295	(64.5)	< 0.001
No	51/149	(35.0)	
Child slept under net last night			
Yes	215/395	(55.6)	0.561
No	20/49	(49.7)	
Household wealth quintile			
1 (lowest)	48/92	(65.2)	0.189
2	50/89	(64.0)	
3	40/88	(58.0)	
4	48/94	(61.7)	
5 (highest)	49/81	(75.3)	

^a^With Rao-Scott correction to account for complex survey design

### Abstracted data

**Fig. 3:**
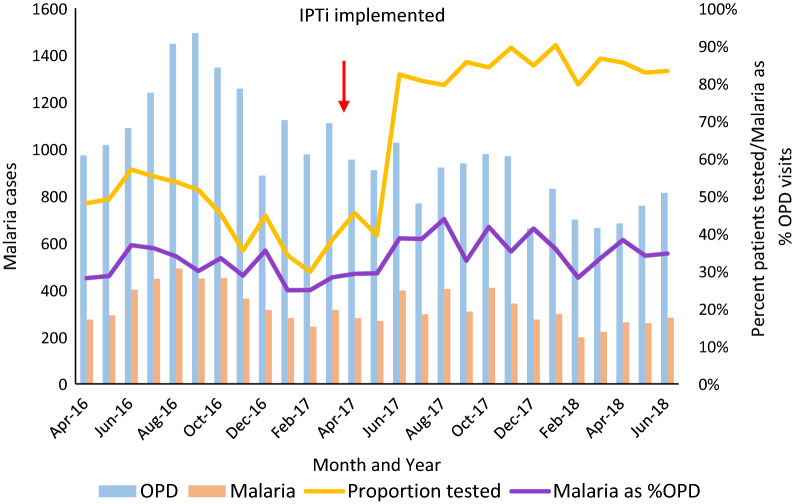
Outpatients, confirmed malaria cases, percent of outpatients tested for malaria, and malaria cases as a percent of outpatient visits, among patients < 12 months in Kambia facilities (n = 17). One facility with data abstracted from registers had more than six consecutive months of missing data and was dropped from the analysis. *OPD* Outpatient department visits

Of the 18 HFs for which data was abstracted from the outpatient and EPI registers, one HF had more than six consecutive months of missing data and was dropped from the analysis. Register data indicated no change in the number of outpatient visits or malaria cases among patients < 12 months old after implementation of IPTi at those HFs (see Fig. 3 and Additional file 2). The proportion of patients < 12 months old who were tested for malaria, a variable significantly related to the number of malaria cases, also increased substantially during this time, from an average of 45% between April 2016 and May 2017 to 78% from June 2017 to June 2018.

### SP resistance markers

**Fig. 4:**
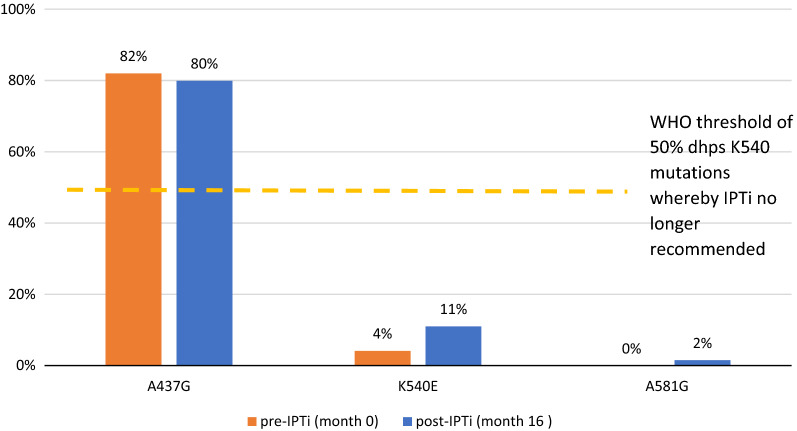
Proportion of parasites alleles with *dhps* mutation

A total of 151 and 153 DBS from RDT-positive patients with similar gender and age distribution were collected from two HFs at pre-IPTi and post-IPTi, respectively. Figure 4 presents the proportion of parasite alleles with *dhps* mutations in both phases. There was no significant difference in the proportion with A437G mutation (82% pre-IPTi [95% CI 77%—86%], 80% post-IPTi [95% CI  76%–85%]; p = 0.84). However, the proportion of parasite alleles with the *dhps* K540E and A581G mutations marginally increased between phases 1 and 2 in samples from the two clinics in Kambia (K540E: 4% pre-IPTi [95% CI 2–7%] vs 11% post-IPTi [95% CI 8–15%], p < 0.01; A581G: 0% pre-IPTi [95% CI 0–1%] vs 2% post-IPTi [95% = 0–3%], p = 0.06). The proportion of parasite alleles with *dhfr* mutations N51I, C59R, and S108N remained fixed at 100% in both phases.

## Discussion

Despite the WHO recommendations for IPTi in malaria-endemic countries where SP resistance levels are not high, Sierra Leone is the first and only country to have introduced and scaled up IPTi nationally through the primary health care system [1]. This evaluation demonstrated how the district of Kambia was able to introduce and successfully maintain IPTi services for at least 16 months. The results showed that HWs were adequately trained; services, supplies and systems were available at the HFs; and a strong community education strategy facilitated community acceptance. To maintain and improve the quality of IPTi services going forward, refresher trainings to the HF staff as well as continued supervisory visits by the DHMTs will be essential to ensure adequate IPTi administration to all eligible infants as well as availability of IPTi supplies and M&E registers. In addition, DHMTs and the NMCP should pay particular attention to closing the gap between IPTi coverage and EPI vaccinations given at the same time.

According to HWs, IPTi was well accepted among caregivers and community leaders. A qualitative assessment in Phase 1 that included focus group discussions with community leaders, community health workers, and caregivers showed that IPTi was generally well accepted in the community and emphasized the importance of the community education strategy and the distribution of promotional materials to ensure community acceptance of IPTi prior to implementation [27, 28]. These findings were also aligned with prior reports that, in trial settings, IPTi delivered together with EPI was well accepted by the communities [29–31].

While this evaluation indicated that Sierra Leone was able to integrate IPTi within the routine EPI program in Kambia, the results highlighted gaps in uptake, especially when compared to other vaccines given at the same visits. While the design of this evaluation did not identify the concrete reasons for these differences in coverage between IPTi and routine EPI, there are some plausible explanations. One reason could be due to staff turnover or training issues. Reasons most frequently reported among caregivers whose children had never received IPTi included not knowing it was required and that their child was not offered IPTi, among others. Most HW reported having administered IPTi to all eligible infants they saw in the past week when asked in phase 1, but it is possible that compliance might have reduced over time or that new HW might have joined after the trainings. For these reasons, refresher trainings among HWs to ensure all children who attend a routine visit receive IPTi are essential to improve future uptake of IPTi.

Another explanation could be that children are vaccinated outside of routine EPI, through catch up, when IPTi is not offered. In fact, the difference in coverage with routine vaccines were particularly concerning for the 9-month visit, with only 36% receiving the 3^rd^ IPTi dose and 50% receiving the first dose of the measles vaccine. Due to recent measles outbreaks in Sierra Leone, the Ministry of Health has supplemented routine measles immunizations with campaigns and outreach services, which could explain the large differences observed as the evaluation was not able to distinguish immunizations provided at the facility or at the community level [32]. In fact, a measles and rubella vaccination post-campaign coverage survey conducted in 2019 identified that in Kambia, 25.8% of children received the measles and rubella vaccine during the campaign instead of through routine services, the highest proportion in the country [33]. These vaccination campaigns could present an opportunity to administer catch up doses of IPTi and reduce the coverage gap.

The success of IPTi relies on a strong EPI program. Despite high acceptance of IPTi among HWs, community leaders, and caregivers, household survey results indicated room for improvement in IPTi coverage—to close coverage gaps with other immunizations given at the same time—as well as ideally, to increase coverage of both IPTi and EPI vaccines. Given the low immunization coverages in Sierra Leone [14], innovative approaches to increase IPTi and EPI vaccines coverage are urgently needed. Building on the strong community health workforce in country, community distribution or defaulter tracing through community health workers could help reduce the coverage gap [34, 35]. Expanding EPI outreach days in the community to address access issues could also address some of the reasons noted for not receiving IPTi or EPI vaccines, as previously suggested [36]. In addition, reinforcing or reinvigorating IPTi messaging at community levels could help increase ITPi coverage. The findings showed that awareness of IPTi was strongly associated with IPTi receipt. However, the most common source of information was from government clinic/hospital, so reverse causality (i.e., that the knowledge of IPTi was due to IPTi administration) cannot be ruled out. Finally, use of enhanced mHealth platforms at health facilities and at the community level could be considered to improve data documentation and help with defaulter tracking [37].

IPTi is recommended in areas where levels of parasite resistance are low (i.e., prevalence of ≤ 50% of the *dhps* K540E mutation) [2], and as such, it is important to monitor SP resistance levels before and after IPTi implementation. This evaluation observed low levels of mutations associated with SP resistance 16 months after the implementation of IPTi with SP in Sierra Leone. A recent review of 12 IPTi trials that enrolled over 19,000 infants observed an overall reduction in clinical malaria of 27% [38]. However, among 10 of the studies using IPTi with SP from 1999 to 2013, there was a slight decline in efficacy in studies conducted after 2009 compared to those conducted before [38]. As the authors describe, this could be due to increased drug resistance, highlighting the need to continually monitor SP resistance markers after IPTi implementation.

One of the strengths of this evaluation is that it was conceptualized prior to IPTi roll-out at the national level in Sierra Leone. By focusing on one of the first districts implementing IPTi, it provided important insights to improve implementation and national scale-up. As the only country to scale-up IPTi at the national level, Sierra Leone’s experience provides valuable information to other countries considering IPTi implementation.

One of the limitations of this evaluation is that it was not able to identify the exact reasons why IPTi coverage was below that of routine EPI. While some plausible explanations are presented, additional research will be needed to address this coverage gap and ensure the success of the national IPTi programme. Also, the evaluation was not able to directly assess if IPTi implementation had an effect on reducing malaria incidence among children in Kambia, as previously demonstrated [3–9]. The evaluation relied on pre- and post-implementation programme data from outpatient and EPI registers to evaluate the effect of IPTi in reducing infant morbidity and mortality, which can be confounded or distorted by different factors such as changes in interventions or issues with data quality or reporting. For example, testing rates at HFs for malaria among patients < 12 months, which is significantly related to the number of malaria cases, nearly doubled around the same time as IPTi introduction, confounding this analysis. This increase in the number of malaria tests may have been related to overall improvement in data collection and completeness in the health facilities during this period.

Additionally, given the modest reduction of 30% in malaria cases in well-designed trials with high coverage [39], the effect size from this programmatic intervention with substantially lower coverage is likely to be much smaller and thus harder to detect using routine data. Another limitation is that the samples for SP resistance testing were taken from symptomatic individuals attending health facilities. While they do not represent a random sample of the population of parasites in the community, presumably the same or similar parasites are circulating among asymptomatic household members and those who become sick with malaria and present to health facilities. Finally, while this evaluation was conducted only in one district in Sierra Leone, it provides important considerations to other countries considering implementing IPTi services routinely.

## Conclusion

According to the 2020 World Malaria Report, based on current trends, many countries will not meet the WHO Global Technical strategy’s 2020 targets to reduce malaria incidence [1, 40]. As such, efforts to improve and expand core interventions and innovative approaches to decrease the burden of malaria, including IPTi where appropriate, are urgently needed. This evaluation demonstrated how one district in Sierra Leone was successful in implementing of IPTi. While gaps in coverage for IPTi and routine immunizations persist in the Kambia district in Sierra Leone, it provides promising perspectives and lessons learned for countries considering implementing IPTi.

## Supplementary Information

**Additional file 1:** IPTi knowledge questionnaire.

**Additional file 2:**Interrupted time-series analysis of confirmed malaria cases among patients <12 months old from register data abstracted from health facilities in Kambia (n=17).

## Data Availability

The datasets used and/or analysed during the current study are available from the corresponding author on reasonable request.
