# Supplementary material for: Evaluation of health system readiness and coverage of intermittent preventive treatment of malaria in infants (IPTi) in Kambia district to inform national scale-up in Sierra Leone
Source: Malar J. 2021 Feb 6;20:74. doi: 10.1186/s12936-021-03615-3 (PMC7866768; doi:10.1186/s12936-021-03615-3)
Supplement: Supplementary file 1 — Additional file 1: IPTi knowledge questionnaire. [file 12936_2021_3615_MOESM1_ESM.docx]

**Additional files**

Additional file 1. IPTi knowledge questionnaire

1. How many doses of IPTi medication should each infant receive in total?
   1. One
   2. Two
   3. Three*
   4. Four
2. At what age should the first dose of IPTi be prescribed to infants?
3. At birth
4. At 4-6 weeks
5. At 8-10 weeks*
6. At any time before the first birthday
7. At what age should the second dose of IPTi be prescribed to infants?
   1. At 6-8 weeks
   2. At 12-14 weeks*
   3. At 6 months
   4. At 10 months
8. At what age should the third dose of IPTi be prescribed to infants?
   1. At 12-14 weeks
   2. At 6 months
   3. At 9 months*
   4. At one year
9. If a nine-month old infant is seen at the facility, and has not received any IPTi medications to date, which of the following is the right thing to do?
   1. Give one dose of IPTi at this visit, and no additional IPTi *
   2. Give three doses of IPTi at this visit
   3. Do not give IPTi
   4. Give one dose at this visit, and ask the parent/caregiver to bring the infant back for two additional doses
10. Is a rapid diagnostic test for malaria routinely required prior to prescribing IPTi?
    1. Yes
    2. No*
    3. Don’t know
11. Which drug should be prescribed to infants for IPTi?
12. Sulfadoxine Pyrimethamine (SP) *
13. Cotrimoxazole
14. Quinine
15. Coartem
16. What is the correct dose of SP for infants weighing less than 5 kg?
17. ¼ of a SP tablet*
18. ½ of a SP tablet
19. 1 SP tablet
20. 2 SP tablets
21. What is the correct dose of SP for infants weighing at least 5 kg?
    1. ¼ of a SP tablet
    2. ½ of a SP tablet*
    3. 1 SP tablet
    4. 2 SP tablets
22. True or false: SP should be crushed and/or dissolved in water and then given to the infant
    1. True*
    2. False
23. Can IPTi be safely dispensed on the same day an infant receives a vaccine?
    1. Yes*
    2. No
    3. Don’t know
24. Can IPTi with SP be prescribed to an infant who has been treated for malaria with an ACT-based drug in the past month?
25. Yes*
26. No
27. Don’t know
28. Should IPTi be prescribed to an infant with acute malaria, e.g., malaria diagnosed on the day of the visit?
    1. Yes
    2. No*
    3. Don’t know
29. Can IPTi be safely dispensed to an infant who had taken another sulfa-based medication (e.g., cotrimoxazole) within the last month?
    1. Yes
    2. No*
    3. Don’t know
30. Can IPTi be safely dispensed to an infant who has not eaten in the past three hours?
    1. Yes*
    2. No
    3. Don’t know
31. What percentage of infants will have allergic reactions to SP?
    1. < 1%*
    2. 1-5%
    3. 6-10%
    4. > 10%
32. For how long is an infant protected from malaria after s/he receives a dose of IPTi?
    1. One week
    2. 30-35 days*
    3. Three months
    4. Six months
33. Where should IPTi services be documented?
    1. The under 5 card
    2. The clinic register
    3. Both A and B*
34. If the child vomits within 30 minutes of receiving SP, what should you do?
35. Send the child home and note this on the <5 card.
36. Immediately administer another dose of IPTi and note this on the <5 card.
    1. Wait 30 minutes, then administer another dose of IPTi and note this on the <5 card. *
37. Other (note what HW says): ________________________
38. If the child vomits after a repeat dose of SP, after vomiting the first dose, what should you do?

1) Send the child home and note this on the <5 card. *

2) Immediately administer another dose of IPTi and note this on the <5 card.

3) Wait 30 minutes, then administer another dose of IPTi and note this on the <5 card.

4) Other (note what HW says): ________________________
