# Supplementary material for: Evaluation of health system readiness and coverage of intermittent preventive treatment of malaria in infants (IPTi) in Kambia district to inform national scale-up in Sierra Leone
Source: Malar J. 2021 Feb 6;20:74. doi: 10.1186/s12936-021-03615-3 (PMC7866768; doi:10.1186/s12936-021-03615-3)
Supplement: Supplementary file 2 — Additional file 2:Interrupted time-series analysis of confirmed malaria cases among patients <12 months old from register data abstracted from health facilities in Kambia (n=17). [file 12936_2021_3615_MOESM2_ESM.docx]

**Additional file 2.** Interrupted time-series analysis of confirmed malaria cases among patients <12 months old from register data abstracted from health facilities in Kambia (n=17)

|  | Incidence Rate Ratio | (95% Confidence Interval) | p-value |
| --- | --- | --- | --- |
| IPTi | 1.065 | (0.695, 1.63) | 0.773 |
| Time | 0.992 | (0.974, 1.009) | 0.353 |
| IPTi*Time | 0.993 | (0.961, 1.026) | 0.674 |
| Proportion tested | 1.999 | (1.232, 3.245) | 0.005 |
| Number of outpatient department visits | 1.009 | (1.006, 1.012) | <0.001 |
| High season | 1.126 | (0.999, 1.270) | 0.051 |

Note: One facility with data abstracted from registers had more than six consecutive months of missing data and was dropped from the analysis.
